# In vitro comparative quality evaluation of different brands of carbamazepine tablets commercially available in Dessie town, Northeast Ethiopia

**DOI:** 10.1186/s40360-023-00670-1

**Published:** 2023-05-25

**Authors:** Biset Asrade, Ejigu Tessema, Abebe Tarekegn

**Affiliations:** 1 Department of Pharmacy, College of Medicine and Health Sciences, Bahir Dar University, Bahir Dar, Ethiopia; 2 Department of Pharmacy, College of Medicine and Health Sciences, Wollo University, Dessie, Ethiopia; 3 Department of Pharmacy, College of Medicine and Health Sciences, Woldia University, Woldia, Ethiopia

**Keywords:** Carbamazepine, Epilepsy, Assay, Identification, Disintegration, Dissolution profile, Dessie

## Abstract

**Background:**

Good-quality drugs that fulfill the regulatory parameters and are produced per the current good manufacturing practice (cGMP) standards are very critical for the best therapeutic outcomes. However, the variety of branded drugs circulation in the market often put clinicians and pharmacists in a difficult situation of choice due to the possibility of interchangeability among brands, so we should ascertain the quality of the various brands of drugs, available in the drug market. The purpose of the study was to evaluate the quality and physicochemical equivalence of six brands of carbamazepine tablets that are commercially available in Dessie town, Northeast Ethiopia.

**Methods:**

An experimental study design was used**.** Six different brands of carbamazepine tablets were purchased from community pharmacies in Dessie town, Northeast Ethiopia, which were selected using simple random sampling methods. Identification, weight variation, friability, hardness, disintegration, dissolution test, and assay for the content of active ingredients were evaluated according to the procedures described in the United States Pharmacopeia (USP) and British Pharmacopeia (BP), and the results were compared with USP and BP standards. The difference (f1) and similarity (f2) factors were calculated to assess in vitro bioequivalence requirements.

**Results:**

The identification test results revealed that all samples contained the stated active pharmaceutical ingredients and all brands of carbamazepine tablets complied with the official specification for weight variation, friability, and hardness tests. The percentage concentration of carbamazepine was found in the range of 97.85 to 102.09, which met the USP specification of 92% to 108% of the stated amount. Similarly, all samples fulfilled disintegration time (i.e., ≤ 30 min) except brand CA1 (34.183 min) and dissolution tolerance limits (i.e., Q ≥ 75% at 60 min), which was found in the range of 91.673% -97.124%. The difference factor (f1) values were < 15 and the similarity factor (f2) values were > 50 for all the tested brands of carbamazepine tablets.

**Conclusion:**

The present study revealed that all brands of carbamazepine 200 mg tablets met the quality control parameters as per pharmacopoeial specifications except the disintegration test of brand CA1, and could be used each brand interchangeably to achieve the desired therapeutic effect.

## Introduction

Epilepsy is a chronic neurological disorder that affects 65–70 million people globally. It occurs in both sexes and at all ages, especially in childhood, adolescence, and increasingly with aging [1, 2].

Drug therapy is the mainstay of the treatment; it is estimated that 70% of patients will respond to the medicines prescribed, while the remainder will need surgery and other forms of therapy to achieve seizure control. Adverse effects are contributors to poor drug compliance, which can be as high as 30–50% of adults living with epilepsy, resulting in a low quality of life and dropouts from drug therapy [3].

**Fig. 1:**
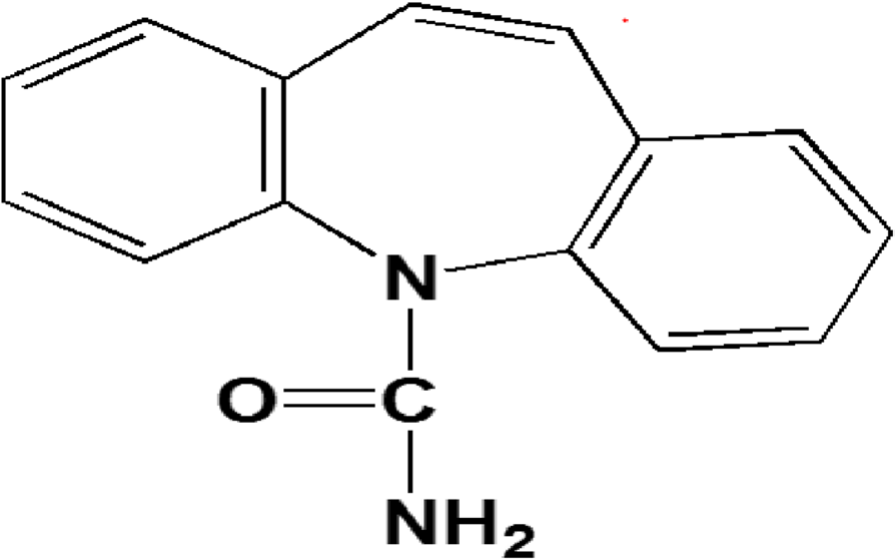
Chemical structure of carbamazepine

Carbamazepine presents a structure related to that of tricyclic antidepressants (imipramine and desipramine) (Fig. 1). It is a white or nearly white crystalline powder; solubility: very slightly soluble in water; freely soluble in acetone and ethanol (96%) [4]. The use of carbamazepine includes the treatment of focal-onset and generalized-onset tonic-colonic seizures, trigeminal neuralgia, and as a mood stabilizer for bipolar disorder treatment. The first-line anti-epileptic drugs like phenobarbital and phenytoin have similar efficacy to some other commonly used anti-convulsion drugs such as carbamazepine and valproic acid but are less expensive to buy, and therefore carbamazepine is the most cost-effective anti-epileptic drug [5, 6].

In Ethiopia the prevalence of epilepsy is 64 per 100,000 population, due to the higher prevalence of epilepsy in Ethiopia, carbamazepine tablets are widely used in Ethiopia, with several new brands having been introduced into the Ethiopian market in recent years [7]. The introduction of generic drug products from multiple sources into the healthcare delivery system of many developing countries has been accompanied by a variety of problems, of which the most critical is the widespread distribution of falsified and substandard drug products [5, 8, 9].

Good-quality drugs that fulfill the regulatory parameters and are produced per the current good manufacturing practice (cGMP) standards are very critical for the best therapeutic outcomes. Therefore, bioequivalence studies become important to assist in the substitution of branded innovator products with generics for affordability while maintaining therapeutic efficacy, which is essential to ensure the absence of any significant difference in the rate and extent to which the active ingredients become available at the site of drug action when administered under similar routes and conditions. Generic drug products must satisfy the same standards of quality, efficacy, and safety as those applicable to innovator products [10–13]. Due to this, the variety of drugs circulation in the market often puts clinicians and pharmacists in a difficult situation of choice because of the possibility of interchangeability among brands, so we should ascertain the quality of the various brands available in the drug market [5, 11, 13]. Hence, this study aimed to determine the quality and the physicochemical equivalence of carbamazepine tablets commercially available in Dessie town, Northeast Ethiopia, and the findings of this research provide more information about the quality and bioequivalence differences among different brands of carbamazepine tablets marketed in Dessie town, Northeast Ethiopia.

## Materials and methods

### Study area and period

This study was conducted in community pharmacies in Dessie town, which is located 401 km to the north of the national city of Addis Ababa and 461.7 km to the west of Bahir Dar. According to the 2007 census, the total population was estimated at 151,174 at the current population density. There are two governmental and three private hospitals, as well as seven governmental health centers, twenty pharmaceutical wholesalers, and thirty-six pharmacies in the town. The study was conducted from February 2021 to July 2021. The reason for selecting this study area was due to the high density of the population, and the availability of a large number of community pharmacies, drug stores, wholesalers, and private health organizations (hospitals and clinics), many clients have access to both prescribed and non-prescribed (OTC) drugs in the study area.

### Equipment and apparatus

The following equipment and apparatus were used for the experiments: High-Performance Liquid Chromatography (HPLC) (H605027, Ultimate 3000, USA, Thermo fisher), UV–Vis spectrophotometer (EVOLUTIN201, Thermo Fisher, USA), analytical balance (MS205DU), pH meter (Mettler Toledo USA), hardness tester (YD-20KZ), friability tester (FT2000SE, Trianda-Tianfa, China), disintegration apparatus (ZB-1E, Trianda-Tianfa, China), dissolution tester (ZRS-8G, China), water bath, beaker, filter paper (Xin Xing China), Hot oven (DHG-9070, Shanghal-Yiheny China), Quartz cuvette, different size flask, pipettes (Pyrex, USA), measuring cylinders, mortar pestle, aluminum foil, Shaking incubator (THZ-300, Shanghi-Yihend, China), and digital caliper (Xin Xing China).

### Chemicals, reagents, and solvents

Analytically graded chemicals, reagents, and solvents were used throughout the experiment process. The solvents and chemicals used in this research experiment were**:** acetonitrile (Sisco RL Pvt. Ltd., India), HPLC-grade ethanol (99.9%) and methanol, formic acid, triethyl amine, diluted phosphoric acid, hydrochloric acid, tetrahydrofuran (LOBA Chemie Pvt. Ltd., India), dibasic sodium phosphate and sodium lauryl sulfate (SCR, China), and deionized water. The reference standard carbamazepine table was obtained from the Ethiopian Food and Drug Authority (EFDA).

### Sampled brands of carbamazepine

All brands of carbamazepine tablets were purchased from a selected private pharmacy in Dessie town, northeastern Ethiopia, which is selected using a simple random sampling method and were randomly coded as CA1 to TE6. Each product was purchased with its original packaging, within its expiration dates, batch number, manufacturer, and country of origin (Table 1). An experimental study design was used and the experiment was done at the quality control laboratory department of Human well Pharmaceutical P.L.C. located in Amhara region, North Shoa, Ethiopia.

**Table 1:** General description of brands of carbamazepine (200 mg) tablets included in the study

Code	Brand name	Batch/lot no	Mfg. date	Exp. Date	Country of origin	Manufacturer
CA1	Calaman	04,201,204	12/2020	12/2022	Ethiopia	Humanwell pharmaceutical
CA2	Carebama	BJW66	4/12/2020	4/2022	Kenya	Dawa limited
CA3	Carma	065,201,200	05/2020	11/2022	Ethiopia	Dukum
CA4	Carzepam	9,060,073	3/2019	07/2021	Ethiopia	E.pharm
ME5	Mezacar	2,002,198	03/20	02/2023	India	Kusum Healthcare Pvt.ltd
TE6	Tegretol	THK45	01/2021	12/2022	Switzerland	Novartis

### Quality assessment parameters

The six brands of carbamazepine 200 mg tablets collected from the study area were tested for identification, uniformity of dosage units, friability, hardness, diameter, thickness, disintegration time, dissolution and assay according to procedures described in United States and British Pharmacopeia [14, 15] in triplicate analysis (*n* = 3), and the average value was used to report the data.

#### Visual inspection

A visual inspection of the uniformity of shape and uniformity of colour, no physical damage, the manufacturer’s address, the manufacturing date, the batch number, the country of origin, the expiry date, cracks, packaging, and the labeling information were checked using the modified World Health Organization (WHO) checklist [16].

#### Identification test

The identification test was done using HPLC, with the retention time of the major peak in the chromatogram of the assay preparation corresponding to the chromatogram of the standard preparation as obtained in the assay [14].

#### Weight variation test

Twenty tablets of each brand were randomly selected and weighed individually on an analytical balance, and their average weight was determined. The percentage deviation from the average was calculated using the Eq. (1) [14]:

1 $$\mathrm{\%\,Deviation}=\frac{\left(\mathrm{Tablet\,weight }-\mathrm{Average\,weight\,Tablet}\right)}{\mathrm{Average\,weight}}X 100$$

#### Friability test

Friability is the measurement of the tendency of tablets to crack, crumble, or break when compressed. Twenty tablets from each brand were randomly selected and weighed initially before undergoing a friability test on an analytical balance. The tablets were placed in the drum of the friability tester and subjected to rotation at 25 rpm for four minutes (100 times). After the procedure was completed, ten tablets were dedusted and weighed. The weights were compared with their initial weights, and then the percentage friability was calculated. According to the USP, the weight loss should not be more than 1%. The percentage of weight loss was calculated using Eq. (2) [14]:

2 $$\mathrm{\%\,Friability}= \frac{(\mathrm{Initial\,weight }-\mathrm{ final\,weight})}{\mathrm{Initial\,weight}}X 100$$

#### Hardness test

The hardness of each tablet was determined by selecting ten tablets randomly from each brand using a hardness tester. Each tablet was placed between two anvils, and force was applied to the anvils. The crushing strength that causes the tablet to break was recorded, and then the mean crushing strengths were determined using Eq. (3) [15, 17]:

3 $$\mathrm{Average\,Hardness }= \frac{\mathrm{Total\,hardness\,of\,all\,tablets}}{\mathrm{Number\,of\,tablet}}$$

#### Disintegration time

The disintegration time was carried out to determine the time required for the tablet to disintegrate. Six tablets were placed in a disintegration tester filled with distilled water and heated to 37 ± 0.5 ^0^C. The tablets were considered completely disintegrated when all the particles passed through the mesh, and the time was recorded [14, 18].

#### Dissolution test

A total of 900 mL of dissolution medium (1% sodium lauryl sulfate (SLS) and distilled water) was used in the dissolution test, which was performed in a USP rotating paddle apparatus at 75 rpm and a constant temperature of 37 ± 0.5 °C. Six samples were weighed and placed in a dissolution tester; each brand was aqueous and analyzed at 5 ml. Samples were withdrawn at 5, 10, 15, 30, 45, and 60 min at the wavelength of maximum absorbance at 230 nm [14].

### Standard preparation

Five mg of USP carbamazepine reference standard was accurately weighed and transferred to a 25-mL volumetric flask, to this, 15 mL of mobile phase was added and mechanically shaken until dissolution was complete. Diluted to volume with mobile phase and mixed at a carbamazepine concentration of 0.2 mg/mL [14].

### Sample solution

The amount of carbamazepine dissolved in 5 ml of sample solution was determined via UV–Visible spectrophotometer at 239 nm in comparison with a standard solution using a 1.0-cm quartz cell and the medium as a blank, and the result was calculated using Eq. (4) [14]:

4 $$\mathrm{Result}= \left(\mathrm{Au}/\mathrm{A s }\right) \times \left(\mathrm{Cs}/\mathrm{L}\right) \times \mathrm{V}\times 100$$

where: Au = absorbance of the sample solution; AS = absorbance of the standard solution; Cs = concentration of the standard solution (mg/mL); V = volume of the medium; L = label claim (mg/tablet).

#### Assay test

The assay test for carbamazepine was done using high-performance liquid chromatography. The liquid chromatography was equipped with a 230-nm detector and a 4.6-mm × 25-cm column that contains packing L10. The flow rate is about 1.5 mL per minute using a mobile phase: a 1000-mL mixture of water, methanol, and tetrahydrofuran (85:12:3) was added, and 0.22 mL of a formic acid mix and 0.5 mL of triethylamine were added [14].

##### Standard preparation

An accurately weighed quantity of USP carbamazepine reference standard was dissolved in methanol and diluted quantitatively with methanol to obtain a solution having a known concentration of about 2 mg per mL. Then, 5.0 mL of this solution was transferred to a 50-mL volumetric flask and diluted with a mixture of methanol and water (1:1) to volume [14, 16].

##### Sample solution preparation

One hundred mg of carbamazepine was accurately weighed and transferred to 50 mL volumetric flasks, dissolved, and diluted with methanol to volume. From this solution, 5.0 mL was transferred to a 50-mL volumetric flask, and dissolved and diluted with a mixture of methanol and water (1:1) to volume [14].

### Data quality control

Quality of experimental results was assured by performing system suitability tests and by strictly applying the procedures as described in the specified monographs of the pharmacopeia.

### Data processing and analysis

The data were collected, checked for completeness, summarized, and tabulated. The analysis was done using Microsoft Excel 2007 and SPSS version 20. Comparison and statistical significance were determined by one-way analysis of variance (ANOVA). A model independent mathematical approach was also used to compare the dissolution profiles of the samples and the reference product using difference factor (f1) and similarity factor (f2). All data were analyzed at 95% confidence interval and P < 0.05 was considered as statistically significant.

## Results and discussion

Evaluating the quality of medicines circulating in the market is important to reduce the risk of having poor-quality medicines in the supply chain. In this study, we assessed the pharmaceutical quality of commonly available brands of carbamazepine tablets in Northwest Ethiopia. All carbamazepine brands were subjected to a number of quality control tests in order to assess their dissolution profile and interchangeability.

### Visual inspection

The physical inspection characteristics of the studied tablet batches showed that all of them had a uniform white color, were undamaged, and did not have any odor. The packaging and labeling of all brands meet the WHO minimum requirement for the packaging and labeling of pharmaceuticals, indicating that the tested samples do not show any signs of being falsely labeled, falsely packed, or falsified products [16].

### Identification test

The retention time of the standard carbamazepine was 0.775 min, and the sample retention time of carbamazepine 200 mg tablet brands were: CA1 (0.770 min), CA2 (0.770 min), CA3 (0.775 min), CA4 (0.775 min), ME5 (0.772 min), and TE6 (0.773 min), which were all in the range of 0.770 to 0.775 min. The retention time of the major peak in the chromatogram of the assay preparation corresponds to the chromatogram of the standard preparation. Therefore, the present study indicated that all brands of carbamazepine tablets contained the stated active pharmaceutical ingredients [14].

### Weight variation test

It is the most significant because it has a relationship with the content uniformity of solid dosage forms. Consequently, the weight variation of the individual tablet is a valid indication of the variation corresponding to the drug content. The average weight of all brands of carbamazepine tablets was found to be between 130 and 324 mg, and it’s percent deviation was less than 7.5 (Table 2). According to USP specifications, all brands met the weight variation test specification [14].

**Table 2:** Results of weight variation, hardness, friability, and disintegration test of different brands of carbamazepine tablets (Mean ± SD)

Brand code	Weight variation (g) *N* = 20	Friability test (%) *N* = 20	Hardness test (N) *N* = 10	Disintegration test (min) *N* = 6
CA1	0.400 ± 6.648	0.402	110.09 ± 19.139	34.183 ± .28
CA2	0.347 ± 5.904	0.001	140.94 ± 7.739	2.4 ± 0.8
CA3	0.276 ± 2.594	0.362	75.16 ± 7.454	1.18 ± 0.18
CA4	0.302 ± 2.594	0.163	65.34 ± 6.107	1.23 ± 0.29
ME5	0.302 ± 5.414	0.0664	104.46 ± 11.558	1.7 ± 0.64
TE6	0.277 ± 4.112	0.179	92.54 ± 7.624	1.33 ± 0.38

### Friability test

Friability was the measurement of the tendency of the tablet to crack, crumble, or break when compressed. This tendency was usually toward uncoated tablets and surfaces during handling or subsequent storage. The mean friability results of carbamazepine tablets were between 0.001% and 0.402%. All brands' results were less than one, hence each brand meets the USP friability specification (Table 2). Therefore, the tablet is less friable, it will maintain a good appearance during storage, transporting, and dispensing, and it has good patient acceptability. Because the patient may get the exact amount of dose and an ultimate treatment outcome may occur [14, 19].

### Hardness test

This is used to ensure the quality of the tablet, and it is an important parameter since tablets must have sufficient ability to survive the handling forces during packaging and breakage under the conditions of storage and transportation. The carbamazepine brands hardness test results were CA1 (110.09 ± 19.139), CA2 (140.94 ± 7.739), CA3 (75.16 ± 7.454), CA4 (65.34 ± 6.107), ME5 (104.46 ± 11.558), and TE6 (92.54 ± 7.624); all brands met the hardness test specification (Table 2). If the hardness of the tablet exceeds a certain limit, which ultimately affects the bioavailability and also influences friability and disintegration time, which means the less hard a tablet, the more friable and the less time it takes to disintegrate [15, 20].

### Disintegration time

Disintegration is a process in which tablets are broken up into granules or smaller particles and is the first step towards dissolution. For a tablet to become fully available for absorption, it must first disintegrate and discharge the drug into the body's fluids [21]. The average disintegration time of all brands of carbamazepine tablets except CA1 was within 30 min, which fulfilled the USP specification. Brand CA3 had the shortest disintegration time (1.18 min). However, brand CA1 had the highest disintegration time (34.183 min), which takes more time to break down into smaller particles because the tablet is too highly compressed (Table 2). Except for CA1, the other brands were adequately compressed during manufacturing with the right amount of the necessary excipients. However, the different disintegration times between the different brands could be because of different binders or disintegrants used in manufacturing. Therefore, the disintegration time is a crucial part of a drug's therapeutic action, which affects bioavailability [14, 21, 22].

### Dissolution test

Dissolution is considered an important tool to predict in vivo bioavailability and has been used to prove bioequivalence to allow interchangeability. The percentage release of carbamazepine API at 60 min for each brand CA1, CA2, CA3, CA4, ME5, and TE6 was 92.205%, 92.823%, 96.91%, 97.124%, 94.228%, and 91.673%, respectively, which is within the acceptance limit, of NLT 75% (Q) [14]. The highest and lowest percent release concentrations were found in samples CA4 (97.124%) and TE6 (91.673%), respectively (Table 3). The dissolution test results revealed that all the brands met USP dissolution limits. However, there is a difference in the percentage release of carbamazepine of different brands. This difference might be attributed to the difference in excipients used and the difference in the manufacturing process used by various manufacturing industries. Therefore, all brands of carbamazepine tablets have good drug release profiles, which are important for their bioavailability and therapeutic effectiveness [14, 22].

**Table 3:** Results of dissolution and assay test of different brands of carbamazepine tablets

Drug product	Brand Code	Dissolution test (mean ± SD)	Assay (%W/W ± SD)
	CA1	92.205 ± 0.102	100.037 ± 2.119
	CA2	98.012 ± 0.062	99.96 ± 1.152
Carbamazepine 200 mg	CA3	96.911 ± 0.067	102.09 ± 0.0483
	CA4	97.124 ± 0.040	99.96 ± 2.38
	ME5	94.228 ± 0.058	102.12 ± 1.057
**Comparator drug**	**TE6**	**91.673 ± 0.047**	97.85 ± 2.093

### Comparison of dissolution profile

**Fig. 2:**
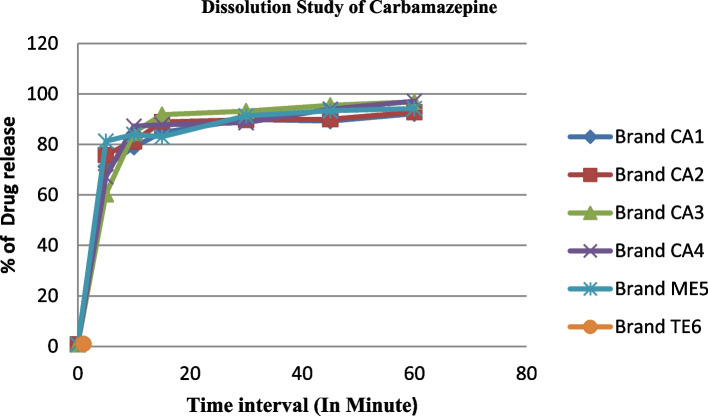
The dissolution profile of different brands of carbamazepine 200 mg tablet in Dessie town

A model-independent approach was used to compare the dissolution profiles of the samples and the reference product, and to ascertain the interchangeability. Difference factor (f1) and similarity factor (f2) have been used frequently for in vitro bioequivalence studies by comparing the dissolution profiles of different brands of pharmaceutical dosage forms with the innovator product [23]. Dissolution testing and consequently comparing the dissolution profiles can be used to establish the similarity of the generic brands to the original product. The f1 and f2 values of all brands of carbamazepine tablets were found in the range of 0.63 to 3.67 and 58.75 to 72.27, respectively, which was found within the specification, between 0 and 15, and 50 and 100, respectively [24]. The result indicated that there was no significant difference between the dissolution profiles of each brand of carbamazepine tablets and that of a comparator drug (Fig. 2). Similarly, Dunnett’s t-test and one-way analysis of variance (ANOVA) at 95% CI showed that the dissolution profiles of each brand of carbamazepine tablets were not significantly different from the innovator product (P > 0.999). Therefore, it will promote the interchangeability of the products to achieve the desired therapeutic effect and to select affordable brands for the patient (Table 4) [24, 25].

**Table 4:** Difference factor (f1) and similarity factor (f2) values of different carbamazepine brands and innovator brands (TE6)

Code	f2	f1
CA1	70.9	3.67
CA2	58.746	1.240
CA3	65.829	0.849
CA4	69.240	0.726
ME5	72.27	0.630
TE6	-	-

### Assay test

The assay determines the concentration of the API found in the sample. The assay test for the carbamazepine 200 mg tablet was done by using the HPLC after conducting a system suitability test. According to USP, an HPLC system is suitable, if the percent relative standard deviation (% RSD) calculated from the peak area obtained from five replicate injections is not more than 2% [14]. The system suitability test results of peak area responses in %RSD was 0.06%. Hence, the system was suitable (Table 5). The percentage concentration of carbamazepine was found in the range of 97.85 to 102.09 (Table 3), which meets the USP specification of 92% to 108% of the stated amount. All the sampled carbamazepine brands passed this test and could be attributed to good manufacturing practices during the preparation of the tablets, which indicates that all the brands contain the required amount of API. Hence, each brand of carbamazepine tablets will not produce an underdose or overdose in the patient [14, 25, 26].

**Table 5:** System suitability test results for chromatographic method of assay of carbamazepine tablets

Standard solution injection	Peak Area	Theoretical plate	Tailing factor	Retention time
1	178.0263	212	1.02	0.777
2	178.1912	211	1.02	0.775
3	178.2799	214	1.02	0.775
4	178.302	216	1.03	0.775
5	178.1813	214	1.03	0.773
Average	178.1961	213	1.02	0.775
SD	0.1088	2	0.01	0.001
RSD (%)	0.06	0.12	0.06	0.15

## Conclusion

The present study revealed that all brands of carbamazepine 200 mg tablets met the quality control parameters as per pharmacopoeial specifications except the disintegration test of brand CA1, and could be used each brand interchangeably to achieve the desired therapeutic effect.

## Data Availability

All data are included within the article.
